# Evaluation of methane mitigation by organic feed additives in dual-flow continuous culture

**DOI:** 10.3168/jdsc.2024-0673

**Published:** 2024-12-19

**Authors:** B.A. Wenner, G. Praisler, K. Mitchell, J. Velez, P.S. Yoder

**Affiliations:** 1 Department of Animal Sciences, The Ohio State University, Columbus, OH 43210; 2 Aurora Organic Dairy, Boulder, CO 80302; 3 Perdue AgriBusiness, Salisbury, MD 21804

## Abstract

Sustainability is interwoven with consumer expectations of organic production systems, yet there are few nutritional options for organic dairy systems to approach methane (CH_4_) mitigation. The objective of the current study was to compare 3 feed additives for CH_4_ mitigation. We hypothesized that each additive would decrease CH_4_ production in continuous culture when compared with a control diet. Using dual-flow continuous culture fermenters fitted for CH_4_ and hydrogen (H_2_) sampling, 4 treatments were arranged in a 4 × 4 Latin square design. Treatments were a negative control (CON, 60:40 concentrate:orchardgrass pellet mix, 17.1% CP, 33.0% NDF, 20.1% ADF, and 27.1% starch) fed twice daily a total 80 g/d DM (CON) and comparing one of the 3 additives: kelp powder (KP) at 1.7 g/d, essential oils (EO) at 3 mg/d, or pistachio-based biochar (BC) at 1.6 g/d. All dosages were calculated based on previous data and supplier recommendations scaled to dual-flow continuous culture functional volume. Experimental periods included 7 d of adaptation and 4 d of sampling (11 d total), and buffer and solids dilution rates were maintained at 7%/h and 5%/h, respectively. The main statistical model included fixed effect of treatment and random effects of fermenter and period. Gas production data were measured by feeding; thus, analysis included a repeated effect of feeding and hourly VFA samples a repeated effect of hour. Only EO decreased CH_4_ production compared with CON (43.1 vs. 47.4 mmol/feeding, SD: 3.96). There was no effect of treatment on H_2_ emission, nor H_2_ or CH_4_ in the aqueous phase. There was also no effect of treatment on nutrient digestibility despite large numerical differences in fiber digestibility, nor the production of primary VFA. However, KP decreased production of isobutyrate and isovalerate compared with CON. While the present data illustrate efficacy of an essential oil product to decrease CH_4_ emission, decreases in CH_4_ were not supported by statistically significant gains in VFA that could translate to increased milk production in the dairy cow.

## Highlights

• Three feed additives were evaluated for the potential to decrease methane emissions.
• The authors used continuous culture to focus on the potential for rumen-specific efficacy.
• Only the essential oil treatment decreased methane without negatively affecting fermentation.
• Continuous culture fermentation is a tool to screen rumen modifiers for ruminal mode of action.

## Graphical Abstract

**Figure:**
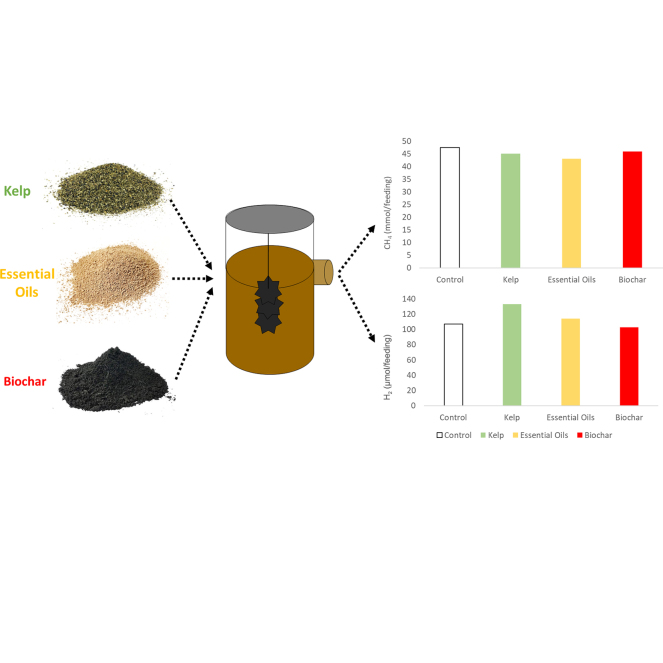
**Summary:** This study aimed to evaluate the ability of 2 commercially available products and 1 product in a developmental stage to decrease methane production within the US organic dairy industry. Dual-flow continuous culture fermenters were fed 1 of 3 different organic feed additives at the recommended dose compared with a control (no additive) in a Latin square design. Only 1 organic feed additive, an essential oil blend, was effective in decreasing daily methane production at modest levels. There were no other effects of note on microbial fermentation or nutrient digestibility. In the current dairy market, decreased methane production without offsetting improvements in nutrient digestibility or utilization must be compensated in some other way in order to warrant implementation on farms.

Enteric fermentation continues to headline discussions of US methane (CH_4_) production despite a relatively steady and apparent low ruminant contribution to the overall carbon footprint in the United States. According to the US EPA (2024), just over 11% of total GHG CO_2_-equivalent emissions are CH_4_, of which approximately 27% are attributed to all of enteric fermentation, which equates to only 3% of US GHG. However, there are still many opportunities to decrease the GHG contribution of the US dairy industry. Among these opportunities, recent feed additives that directly target methanogenesis are coming to market that can potentially decrease enteric CH_4_ emissions from ruminants by a conservative 20% to 30% depending on management situations (Roque et al., 2019; Hristov et al., 2022; Ma et al., 2024). As challenges facing implementation are addressed (Vijn et al., 2020; Hristov, 2024) and additives seek consumer acceptance, the organic dairy industry continues to have far fewer options to decrease CH_4_ emissions through scientific peer-reviewed feed additives.

At the last USDA census, the organic dairy industry in the United States represented ∼3,000 farms and over 300,000 cows, and the organic livestock market continues to grow in sales (USDA, 2020). Thus, it is prudent to explore the efficacy of feed additives that are already permitted for organic feeding and have the potential to decrease CH_4_ emissions. Due to the known issues of many CH_4_ inhibition strategies leading to lost energy or unaccounted hydrogen (H_2_) escape, dual-flow continuous cultures are an ideal system to evaluate new feed additives. This system enables the measure of production of VFA (Li et al., 2022) and gas production (Wenner et al., 2017), with digestibility estimates flawed for high starch digestibility and inverted fiber digestibilities (Hristov et al., 2012), but demonstrated to pair well with on-farm results (Wenner and St-Pierre, 2019).

The objective of this study was to use the dual-flow continuous culture research model to evaluate 3 potential candidates for CH_4_ mitigation in organic production settings. Our hypothesis was that each of the 3 additives would decrease CH_4_ by shifting VFA production pathways to favor propionate or other reduced end products over acetate, or provide an alternative H_2_ sink, decreasing metabolic hydrogen available for methanogenesis.

The present study applied treatments to a 4 × 4 Latin square design using dual-flow continuous culture fermenters (n = 4). Twice daily, all fermenters were fed 40 g DM of a pelleted 60:40 concentrate:orchardgrass pelleted diet (33.0% NDF, 20.1% ADF, 27.1% starch, 17.1% CP, and 2.2% ether extract). The pelleted concentrate was the same design—primarily corn-soybean hulls with added distillers grains and soybean meal—as previously reported by Wenner et al. (2020). Fermenters were provided 1 of 4 treatments: (1) no additive control (**CON**), (2) 1.7 g/d supplemental kelp powder (**KP**; Thorvin, New Castle, VA), (3) 3 mg/d essential oil blend (**EO**; Agolin Naturu, Agolin SA, Bière, Switzerland), or (4) 1.6 g/d biochar from pistachio hulls (**BC**). Products were selected for evaluation based on current availability and scaling for incorporation into the organic dairy market, as well as an intended difference in mode of action across treatments. Doses were determined based on feeding rate recommendations for dairy cows, scaled on rumen volume to the dual-flow fermenter vessel working volume. Dosing of the EO treatment was conducted by dilution of the product at time of dosing into 1 mL of distilled H_2_O that was pipetted below the surface of the culture while agitated.

Each experimental period began by sampling the rumen contents of 2 ruminally cannulated lactating Jersey cows housed at The Ohio State University Waterman Dairy (Columbus, OH) and cared for under Institutional Animal Care and Use Committee protocol #2013A00000073. The donor cows used in this study were not previously fed monensin. Rumen contents were sampled, squeezed through 2 layers of cheesecloth into prewarmed bottles, and transported by insulated cooler at 39°C back to the laboratory for inoculation. Inocula sources were pooled from both cows and added to fermenters at 50% of total working volume. Clarified rumen fluid (centrifuged at 15,000 × *g*, 4°C, 15 min, and then autoclaved) was diluted 1:20 with anaerobic buffer for the first buffer batch (approximately 1.5 d) to better adapt microbial contents to fermenters.

Dual-flow continuous culture fermenters (n = 4; Hoover et al., 1976), with adaptations for protozoal retention (Karnati et al., 2009), improved mixing (Wenner et al., 2021b), and tighter gas sealing with a clamp that is described here for the first time (Figure 1) but was first used in Mitchell et al. (2023). Fermenters were given 7 d of adaptation (5 d on treatment), followed by 4 d sampling period. Fermenter working volume was 1.71 L, stirring set to 50 rpm, temperature set at 39°C, total buffer dilution rate fixed at 7.0%/h for all treatments, and solids dilution rate fixed at 5.0%/h for all treatments. Buffer pH was maintained between 6.70 and 6.75 under continuous bubbling of CO_2_ to maintain anaerobic conditions. Fermenter buffer was made according to Weller and Pilgrim (1974) with 40 mg/dL urea added to buffer to ensure concentrations of NH_3_-N were not limiting.

**Figure 1:**
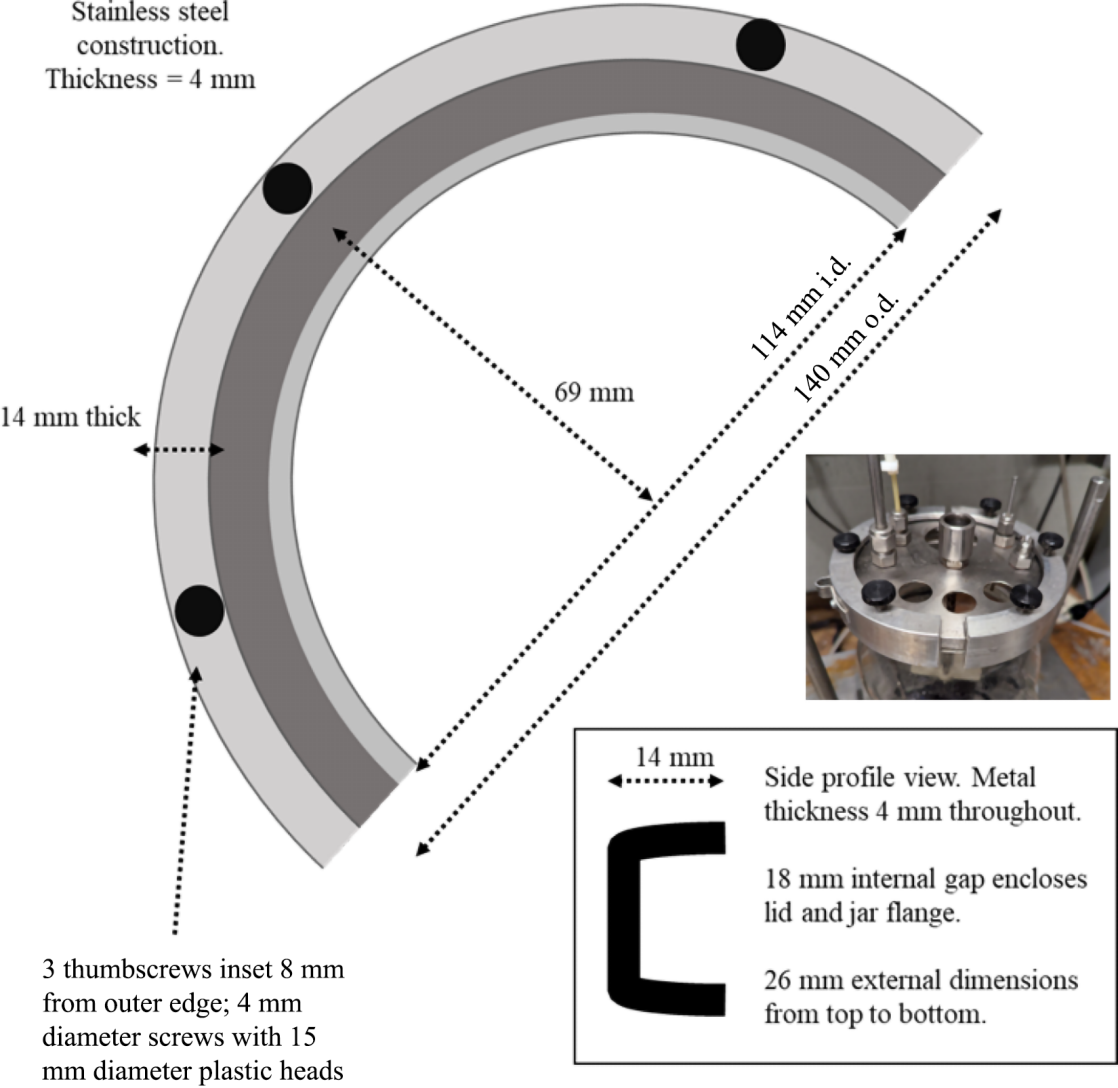
Design and specifications for the “half moon” clamp added to dual-flow continuous culture system previously described by Hoover et al. (1976) and Wenner et al. (2021b). Clamps were first used in Mitchell et al. (2023) and improved sealing and eliminate need for thick lids described in Wenner et al. (2021b).

Both redox (Pinpoint ORP, American Marine Inc., Ridgefield, CT) and pH (Precision pH Meter, ThermoWorks, American Fork, UT) were recorded hourly between d 5 and 8, hourly from 0 to 12 h postfeeding. Daily fermenter effluent was cumulatively collected for 24 h postfeeding in containers on ice from d 8 to 11. A 30 mL subsample was taken and mixed with 3 mL of 6 *N* HCl for VFA and NH_3_ analysis. Two additional 10 mL aliquots were taken and preserved in 50% formalin or at −80°C for protozoal counting and DNA extraction, respectively, and are reported in a parallel article (Park et al., 2024). A subsample representing 20% of the daily effluent was taken and dried at 55**°**C for various analysis; the remaining effluent was discarded. After effluent samples were dried, they were submitted to Cumberland Valley Analytical Services (Waynesboro, PA) for analysis of DM, CP, NDF, ADF, starch, water-soluble carbohydrate (**WSC**), ether extract, and ash. Liquid effluent VFA and NH_3_-N concentrations were determined according to Harvatine et al. (2002) and Chaney and Marbach (1962), respectively.

Fermenter CH_4_ and H_2_ emission was measured using the Micro-Oxymax detection system (Columbus Instruments Inc., Columbus, OH) adapted for the dual-flow continuous culture fermenters as previously described by Wenner et al. (2017) with the exception that CH_4_ was capture per feeding rather than per day. If a sampling device leaked during the 12-h interval, the interval was discarded, resulting in 7 complete intervals per treatment per period. Sensors were calibrated with a custom tank (Praxair, Columbus, OH) at the start of every experimental period. Gas production data were also taken in tandem with fermenter liquid effluent samples used for VFA and aqueous phase H_2_ [**H_2_(aq)**] according to Mitchell et al. (2023) and Wenner et al. (2020), respectively, on the last 4 d of each experimental period at 0, 0.5, 1, 2, 4, and 8 h after feeding for the morning feedings only. Samples for VFA were pooled across days for the same time point.

Nutrient digestibilities, VFA, NH_3_ concentrations, and ^15^N enrichment data were analyzed using the MIXED procedure of SAS 9.4 (SAS Institute Inc., Cary, NC) according to the following model:

*Y_ijkl_* = *µ* + *T_i_* + *p_j_* + *F_k_* + *d_l_* + *Ε_ijkl_*,

where *Y_ijkl_* is the dependent variable, *µ* is the overall population mean, *T_i_* is the fixed effect of *i*th PFA treatment (*i* = CON, KP, EO, BC), *p_j_* is the random effect of *j*th period (*j* = 1, 2, 3, 4), *F_k_* is the fixed effect of *k*th fermenter (*k* = 1, 2, 3, 4), *d_l_* is the random effect of *l*th day (d = 8, 9, 10, 11), and *Ε_ijkl_* is the residual error, assumed independent and $\&sim;N\left(0,\sigma_{e}^{2}\right).$ Data analysis for hourly gas production, VFA proportion, and H_2_(aq) included a repeated statement for hour, whereas daily gas production included a repeated statement day. The covariance structure with the lowest Bayesian information criterion was used—in this case AR(1) and a Kenward-Rogers adjustment for degrees of freedom applied. Significance was declared at *P* < 0.05 and trends for 0.05 ≤ *P* < 0.10.

The pH of the continuous cultures averaged 6.35 at feeding, reaching a nadir of 6.10 at 5 h postfeeding, before recovering for the remaining 7 h until the next feeding; there was no treatment effect on pH (*P* = 0.82, data not shown). Similarly, there was no treatment effect (*P* = 0.55) on culture redox (data not shown), which averaged −315 Eh and is best characterized by a steep decline postfeeding until 3 h followed by a quick recovery (1.5 h) and a steady redox for the remainder of the day. Additionally, it was observed that the BC-treated fermenters were stained black by the treatment and required extensive cleaning.

There was a significant effect of treatment on CH_4_ production with EO supplementation reducing CH_4_ production compared with CON and BC treatments (decreased 9.1%, Table 1, *P* < 0.01). The KP treatment tended to decrease CH_4_ to the CON (lower by 4.9% vs. CON; *P* = 0.07). There was no effect (*P* > 0.10) of treatment on H_2_(aq) or CH_4_(aq), either daily averages (Table 1) or by hourly samples (data not shown), perhaps due in part to the proportionally large variation known to the current method (Wenner et al., 2017). While many in vitro evaluations yield lower CH_4_ than in vivo studies, the current study falls within an estimated 1.0 to 1.3 mmol/g DM fed in a recent publication conducted in chambers (Bica et al., 2022).

**Table 1:** Nutrient digestibilities, gas production, effluent flow, and VFA concentration and production from continuous culture fermenters supplemented with 1 of 3 organic feed additives^1^

Item	CON	KP	EO	BC	SEM	*P*-value^2^
Effluent, L/d	2.86	2.91	2.87	2.92	0.06	0.18
Apparent OM digestibility, %	44.9	45.4	46.5	45.3	2.1	0.94
Apparent starch digestibility, %	99.3	99.7	99.4	99.6	0.3	0.58
Apparent WSC digestibility, %	67.2	73.3	69.2	66.9	4.0	0.67
NDF digestibility, %	39.7	44.7	46.8	43.2	4.5	0.55
ADF digestibility, %	49.8	48.1	52.3	48.0	2.8	0.51
NH_3_-N, mg/dL	11.6	8.9	11.8	12.0	1.0	<0.01
H_2_(aq), μ*M*	4.36	4.62	4.21	4.42	1.17	0.94
CH_4_(aq) μ*M*	109	122	113	113	15	0.94
Daily H_2_ production, μmol/feeding	107	133	114	103	54	0.88
Daily CH_4_ production, mmol/feeding	47.4	45.1	43.1	45.9	4.0	<0.01
CH_4_/NDF digested, mmol/g	9.68	7.50	6.98	7.74	1.32	0.23
Total VFA, m*M*	115	114	116	112	2.8	0.51
Individual VFA, mol/100 mol						
Acetate	59.3	59.4	58.7	59.3	0.6	0.32
Propionate	22.2	23.2	23.4	21.7	0.7	0.34
Isobutyrate	0.701	0.608	0.682	0.732	0.047	<0.01
2-Methylbutyrate	1.45	1.54	1.49	1.41	0.11	0.29
Butyrate	13.9	13.1	13.3	14.4	0.7	0.25
Isovalerate	0.606	0.488	0.591	0.617	0.045	<0.01
Valerate	1.76	1.67	1.84	1.74	0.09	0.07
Caproate	0.777	0.719	0.850	0.795	0.079	0.09
Acetate:propionate	2.68	2.57	2.51	2.74	0.10	0.38
VFA, mmol/d	328	333	334	328	6.1	0.68
CH_4_/total VFA, mmol/mmol	0.281	0.264	0.255	0.274	0.021	0.04

1 Treatments are CON (control diet, no additive), KP (1.7 g/d supplemental dried kelp), EO (3 mg/d supplemental essential oil), and BC (1.6 g/d supplemental pistachio hull biochar).

2 *P*-values reported for the effect of treatment where significance was declared at *P* < 0.05 and trend at 0.05 < *P* ≤ 0.10.

Although there were large numerical differences in fiber digestibility estimates between treatments, nutrient digestibility was not affected by any supplement provided (*P* > 0.10). Apparent starch digestibility was particularly high (>99%) across all samples; this is typical of continuous culture and has been noted previously (Wagner et al., 2018). Ammonia concentration was decreased by KP when compared with the other treatments (by at least 2.5 mg/dL), possibly indicating decreased proteolysis, but none fell outside a recent N source dose-response study in continuous culture (Wenner et al., 2021a). It is interesting to note that apparent WSC digestibility was less than starch digestibility in the present study. To the authors' knowledge, this is one of the first times that WSC has been reported in continuous culture and the differential between WSC and starch digestibility requires additional inquiry in future studies. It is possible that sugar passed with the liquid passage rate due to its greater solubility and this decreased apparent digestibility when compared with starch-heavy particles.

A meta-analysis on the efficacy of the specific EO blend used in the present study reported a decrease CH_4_ emission (−9.9% when component corrected) and improved dairy cow efficiency (+4.4%), with the expectation that a 4-wk adaptation is optimal for full efficacy (Belanche et al., 2020). Methane offset in the current study is in line with this meta-analysis. More recent studies using a commercial version of the EO blend (Agolin) in lactating cows have emphasized decreased CH_4_ intensity rather than net decrease in emissions (Carrazco et al., 2020; Silvestre et al., 2023) and decreased ammonia. An increase in milk component yield without a changing CH_4_ production and an apparent shift in CP degradation collectively point toward a shift in microbial species that promote metabolic efficiency with EO treatment.

It is possible that all treatments could have benefited from a longer adaptation period and further investigation at a longer adaptation period could demonstrate efficacy for KP or BC. However, EO cultures specifically were sequenced daily in a parallel study that described microbiomic consistency being reached at 5 to 6 d postinoculation (Wenner et al., 2023) and similarly in a broader study by Salfer et al. (2018). The lack of a rumen modifier present in the inoculum may have increased the sensitivity of cultures in this study (Weimer et al., 2011) and contributed to more rapid community responses to the treatments. The abbreviated adaptation period in this study versus that of in vivo studies could also indicate either that the decreased methanogenesis may have intensified over time if the study were extended or alternatively indicate that the cultures could become adapted to treatment and lost efficacy as has been observed in several feed additives (Benchaar and Greathead, 2011).

The lack of response in BC is contrary to expectations given that this same biochar was screened in a batch culture study by Way (2020) and found to decrease CH_4_ by 30% at a feeding rate of 2% of dietary DM. A different biochar product was tested in RUSITEC and also found to decrease CH_4_ when included between 0.5% and 1.0% of dietary DM (Saleem et al., 2018), whereas another RUSITEC study found no effect of 3 chemically treated spruce biochars (Tamayao et al., 2021). Further, using a pine-based biochar, Terry et al. (2019) also described lack of efficacy in cattle within respiratory chambers despite a 25% decrease in protozoal populations. In the present study, 2 batches of BC were received before a potential source was chosen and they displayed vast compositional differences: 54% versus 97% DM, 91% versus 96% ADF, and 14% versus 9% ash. It is likely that variation in the production of biochar is the leading limitation to current use of biochar as any other variable. This variation would explain the discrepancy in published literature on the efficacy of biochar for methane mitigation.

Table 1 reports VFA concentrations in daily effluent samples, and there was no effect of treatment on total VFA concentration or production, nor on proportion of acetate, propionate, butyrate, or 2-methylbutryate (*P* > 0.10). Therefore, when considering CH_4_ production scaled to total VFA, there was also a significant decrease by EO treatment (*P* = 0.04). However, KP depressed isobutyrate and isovalerate compared with other treatments (*P* < 0.01) and tended (*P* ≤ 0.09) to decrease valerate and caproate. We speculate that the decrease in ammonia concentration by KP (Table 1, *P* < 0.01) reflects a poorer fermentation profile and less CP degradation, which pairs with the decreased isobutyrate and isovalerate for KP. This pattern carried across time with a consistently lower proportion of both isobutyrate and isovalerate in the KP treatment (data not shown). Alternatively, EO treatment tended to elevate both valerate and caproate versus CON (*P* ≥ 0.09), potentially accounting for a fraction of the metabolic hydrogen offset from decreased CH_4_ emissions. Another continuous culture experiment using a different essential oil blend also saw changes exclusively in valerate (Castillejos et al., 2007); however, in that study valerate was decreased by the lowest dose, whereas Castillejos et al. (2008) observed *increases* in valerate under either monensin or high rosemary oil doses. The only other effect of treatment over time of VFA sampling was that propionate was decreased by BC (*P* = 0.02) at 1 h postfeeding.

An obvious limitation of this study is the lack of microbial growth data useful in building a more complete picture of hydrogen balance. However, the combination of steady H_2_(aq) with increased H_2_-utilizing VFA suggests that the decrease in CH_4_ is likely a response to shifted microbial fermentation pathways at the whole microbiome level and in vivo data in future studies could be used to support that hypothesis. Although 2 of the 3 treatments were not effective in decreasing methanogenesis (KP and BC), these limitations could be related to effective dose or product variability. Castillejos et al. (2007) demonstrated that continuous culture fermenters can be very dose responsive but they can also serve as an effective screening tool to evaluate an additive under adapted conditions rather than using batch cultures highly susceptible and responsive to treatments.

In the present study, only one treatment (EO) was effective in substantively decreasing CH_4_ emissions at the dose suggested by current feeding rates in lactating dairy cows. Improving our knowledge about the mechanism by which this (and other) EO blend decreases methanogenesis is essential to pursuing more effective approaches both within organic and conventional dairy systems. The current dual-flow approach demonstrates a ruminal treatment that effectively decreased methanogenesis in vitro, but more work is needed to validate the sustained effect of the EO treatment, especially in vivo and over long-term lactation studies.
